# Bi_2_W_2_O_9_ Nanoflakes Synthesized via a Hydrothermal Method: Antibacterial Potency and Cytotoxicity Evaluation on Human Dermal Fibroblasts

**DOI:** 10.1021/acsomega.4c07612

**Published:** 2025-02-05

**Authors:** Muthamizh Selvamani, Dilipan Elangovan, Ali Alsalme, Arul Varman Kesavan, Ganeshraja Ayyakannu Sundaram, A. Santhana Krishna Kumar

**Affiliations:** † Department of Physiology, Saveetha Dental College & Hospitals, Saveetha Institute of Medical & Technical Sciences, Saveetha University, Chennai 600077, Tamil Nadu, India; ‡ Department of Chemistry, College of Science, King Saud University, Riyadh 11451, Saudi Arabia; § Department of Physics & Nanotechnology, SRM Institute of Science & Technology, Kattankulathur 603203, Tamil Nadu, India; ∥ Department of Research Analytics, Saveetha Dental College and Hospitals, Saveetha Institute of Medical and Technical Sciences, Poonamallee High Road, Chennai 600 077, Tamil Nadu, India; ⊥ Department of Chemistry, National Sun Yat-sen University, No. 70, Lien hai Road, 17 Gushan District, Kaohsiung 80424, Taiwan; # Department of Chemistry, Saveetha School of Engineering, Saveetha Institute of Medical and Technical Sciences (SIMATS), Saveetha University, Chennai 602 105, Tamil Nadu, India

## Abstract

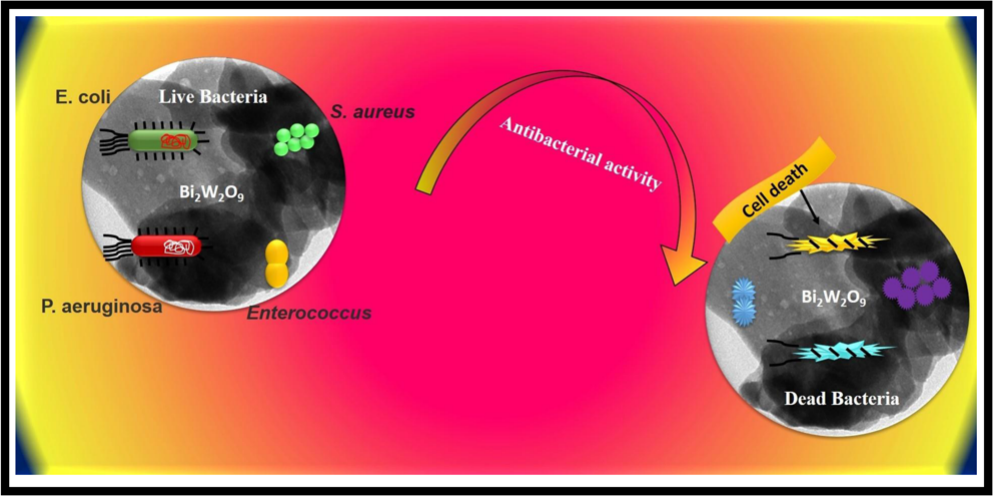

The prevalence of disease and death caused by pathogenic microbes is on the rise, and increasing rates of antibiotic resistance are concerning. This study investigates the antibacterial properties and cell viability (NHDF) behavior of synthesized Bi_2_W_2_O_9_ nanoflakes. The Bi_2_W_2_O_9_ nanoflakes were synthesized using the hydrothermal method, and their physical and compositional stability was analyzed through various characterization techniques, including X-ray diffraction (XRD), X-ray photoelectron spectroscopy (XPS), Fourier transform infrared (FTIR), Raman, and DRS-UV. The Bi_2_W_2_O_9_ nanoflakes demonstrated promising antibacterial properties, with no significant cytotoxic effects, such as cell death or detachment, observed. This study confirms that Bi_2_W_2_O_9_ nanoflakes exhibit antibacterial activity against oral pathogens while maintaining 90% cell viability in normal human dermal fibroblast cell lines, paving the way for new therapeutic options for the treatment of oral infections.

## 1 Introduction

Antibacterial compounds are essential to the food packaging, textile, pharmaceutical, and water disinfection industries.^1,2^ The importance of inorganic disinfectants is increasing due to the negative effects of organic disinfectants, most notably their toxicity to humans. In terms of specificity, toxicity, robustness, and heat resistance, inorganic antibacterial agents perform better than organic antibacterial substances.^3,4^ Despite being a heavy metal, bismuth is safe and does not cause cancer. Because of its safety and effectiveness, scientists and the pharmaceutical industry have become interested in it, which has resulted in the invention of various medications, some of which have already been effectively implemented in clinical procedures.^5^ Bismuth-containing nanoparticles, or Bi NPs, are still in the early stages of development, but because of their remarkable properties—high surface area, excellent stability, affordability, desired catalytic efficiency, ease of functionalization, low toxicity, and potent antibacterial activity—they have recently garnered a lot of interest as a scientific breakthrough with potential applications in medicine.^6^ Bi is also thought to be among the least hazardous and physiologically inert heavy metals when compared with other metals, which makes it a better choice for in vivo applications. Bi NPs also have the advantage of being easily controllable in terms of size and form during synthesis, opening up new possibilities for future therapeutic applications.^7,8^ Bi NPs are compared to other metal NPs, and the aforementioned characteristics have fueled the increased interest in Bi NPs for biological applications. The emphasis of research by several scientists is on microbial infections caused by resistant bacteria, which have led to significant global public health issues. The goal is to develop safer, more effective, and less toxic antimicrobials. One of the most modern treatment methods to restrict and manage bacterial development is the use of nanoparticles (NPs) with antibacterial possessions.^9^ The nanoparticles of metal and metal oxide have been implicated in the majority of findings of this antibacterial activity.^10^ The primary focus of the argument, however, is on the potential harm they may do, namely, the accrual of these metal nanoparticles in various humanoid organs. However, the usage of nonmetallic-related antimicrobial NPs is currently partial despite their potential antibacterial action.^11^ Notably, due to its high similarity in the structure to the extracellular matrix, electrospun nanofibers have been widely used in the biomedical area.^12,13^ A novel family of fluorescent nanostructures known as carbon quantum dots (CQDs) has garnered significant interest because of its intriguing characteristics, which include high quantum yield (QY), photostability, adjustable excitation and emission, low cytotoxicity, and excellent biocompatibility. Although many carbon-based nanomaterials, including fullerenes, single-walled carbon nanotubes, and graphene oxide, have demonstrated strong microcidal qualities, CQDs are not frequently employed as antimicrobial agents.^14,15^ CQDs are one of many carbon-based nanomaterials that have gained interest because of their exceptional biocompatibility, low toxicity, high water solubility, and chemical stability. One of the most prevalent ways that CQDs fight bacteria is by the cation effect, in which positively charged CQDs electrostatically attach to bacteria, breaking down their cell walls and ultimately killing the bacteria. Elements can be added or removed to modify the structures and compositions of CQDs. By altering the electron density of the CQD system, we can give more electron-active sites. Since nitrogen (N) atoms are bonded to carbon atoms by five valence electrons, they are the most researched dopant. It offers a wide range of functionalization choices for CQDs. Phosphorus (P) atoms can also function as n-type donors to modify the structure of CQDs, much like N atoms can. Moreover, sulfur (S) atoms at the nanoscale are widely used as dopants because they are environmentally friendly, water-soluble, and antibacterial.^16^

Little study has been done on polymeric NPs and nanochitosan. It is known that bismuth subsalicylate (BSS) and other bismuth (Bi) compounds are effective against a variety of microbes in the antibacterial arena.^17^ Antimicrobial stuffs of several Bi compounds and BSS NPs generated by laser ablation of solids in liquid media (LASL) have been demonstrated against oral anaerobic bacteria such as *Aggregatibacter actinomycetemcomitans* and *Porphyromonas gingivalis*, as well as aerobic opportunistic pathogenic bacteria such as *Escherichiacoli*, *Pseudomonasaeruginosa*, *Staphylococcusaureus*, and *Staphylococcus epidermidis*.^18^ These germs in in vitro development were successfully inhibited by BSS (bulk or NPs), according to the results. Nevertheless, it has been demonstrated that oral bacteria from clinical samples exhibit unique patterns of sensitivity or even greater resistance than their counterparts in the laboratory, so it is imperative to investigate their susceptibility.^19^ Bismuth has a long history of medical usage; for more than 300 years, syphilis and skin lesions have been treated with bismuth salts.^20^ For example, Louis Odier treated dyspepsia experimentally in 1786 using bismuth subnitrate. Research demonstrating bismuth efficacy against *Helicobacter pylori* (*H. pylori*) has led to a revival of bismuth in modern medicine despite its use declining as antibiotics became more common.^21^ It is simple and noninvasive to harvest dermal fibroblasts from donors or patients. Dermal fibroblasts in humans are capable of transdifferentiating into cells that resemble fat, cartilage, bone, and chondrocytes. Studies in the preclinical and clinical stages have demonstrated that nHDFs implanted in an extracellular matrix derived from human collagen may effectively integrate into the host and promote wound healing following surgery. The previous report on the use of the rabbit disk degeneration model to do a pilot study to investigate if transplanting nHDFs may minimize disk degeneration and inflammation.^22^

Bi_2_W_2_O_9_ nanoflakes were created in the current work using the hydrothermal technique, and their antibacterial and cell viability properties were evaluated against cell lines of normal human dermal fibroblasts (NHDF). The antibacterial activity against *E. coli*, *Enterococcusfaecalis*, *S. aureus*, *P. aeruginosa*, and normal human dermal fibroblast (NHDF) cell lines was tested with the fabricated Bi_2_W_2_O_9_ nanoflakes.

## 2 Materials and Methods

### 2.1 Materials

Analytical rating substances were obtained from Sigma-Aldrich Chemicals, India, for the manufacture of bismuth tungstate, namely, bismuth nitrate (Bi(NO_3_)_3_·5H_2_O) and sodium tungstate (Na_2_WO_4_·2H_2_O). Every chemical was pure for analytical purposes. The solvents were ethanol and double-distilled water. Mueller-Hinton agar was obtained by purchasing representative species of Gram-positive and Gram-negative bacteria, including *E. coli*, *E. faecalis*, *S. aureus*, and *P. aeruginosa*, from Himedia, Mumbai, India, and the American Type Culture Collection (ATCC-25923, ATCC-25922). The ingredients include Dulbecco’s modified Eagle’s medium (DMEM), fetal bovine serum (FBS), 0.25% trypsin EDTA, dimethyl sulfoxide (DMSO), and methyl thiazolyl diphenyl-tetrazolium bromide (MTT).

### 2.2 Synthesis of Bi_2_W_2_O_9_

The reagents utilized in this investigation were all analytically pure and did not require any additional purification. The hydrothermal method was used to prepare Bi_2_W_2_O_9_, and the general synthesis procedures were as follows: sodium tungstate (Na_2_WO_4_·H_2_O) was dissolved in Milli-Q water in appropriate proportions. Following the dissolution of proportionate bismuth(III) nitrate pentahydrate [Bi(NO_3_)_3_·5H_2_O] in nitric acid, the anionic surfactant sodium dodecyl sulfate (SDS) was added to the solution sequence while stirring to create a transparent solution and guarantee that Bi/W stays 1:1 throughout the Bi_2_W_2_O_9_ production process. We used a NaOH solution to bring the pH of the mixture down to about 7. After 30 more minutes of stirring, the slurry was transferred into stainless steel autoclaves lined with Teflon. After that, the sealed reactors were heated for 12 h to 180 °C. After allowing the products to naturally cool to ambient temperature, they were recovered by centrifugation, and after multiple washes in Milli-Q water and ethanol to ensure that any remaining contaminants were eliminated, they were dried for 8 h at 80 °C.

### 2.3 Investigation of Antibacterial Activity by the Well Diffusion Method

Material testing was carried out using the diffusion technique. An alternative concentration of bismuth tungstate (100 μg/mL) was reconstituted using dimethyl sulfoxide (DMSO). The spread plate method was used to deposit 10 μL (10 cells/ml) of the test microorganisms into the suitable medium, together with the 24 h cultures of bacteria cultivated in nutritious broth. A sterile cork borer with a diameter of 5 mm was used to create the wells. Once the wells had been set, the extracts were placed on plates that had been seeded with the test organisms. The antibacterial test standard used in this study was tetracycline (10 μg). The antibacterial test plates were subjected to a 24 h incubation period at a temperature of 37 °C. The diameters of the inhibitory zones were determined in millimeters.^14^

### 2.4 Cell Culture Conditions

Cells with a density of 1 × 10^5^ cells per well were seeded in 96-well plates and placed in a 370 °C incubator with 5% CO_2_ ambiance. Once the cell had reached confluence, the samples were subjected to UV treatment and then transferred onto a 96-well plate for incubation for a duration of 1 to 3 days. Following the incubation period, the sample was extracted from the well and subjected to a washing procedure using either phosphate-buffered saline (pH 7.4) or DMEM without serum. A solution of 0.5% 3-(4,5-dimethyl-2-thiazolyl)-2,5-diphenyl--tetrazolium bromide (MTT) was added at a concentration of 10 μL/well (5 mg/mL) and incubated for a duration of 4 h. Following the incubation period, 1 mL of DMSO was introduced into each well. The ELISA reader was used to measure the absorbance at 570 nm, with DMSO serving as the blank.^15^ The experiment included making measurements and visually determining the concentration necessary to achieve a 50% inhibition, often known as IC_50_.

The percentage of cell viability was determined by using the below formula
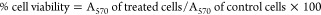


**Scheme 1:**
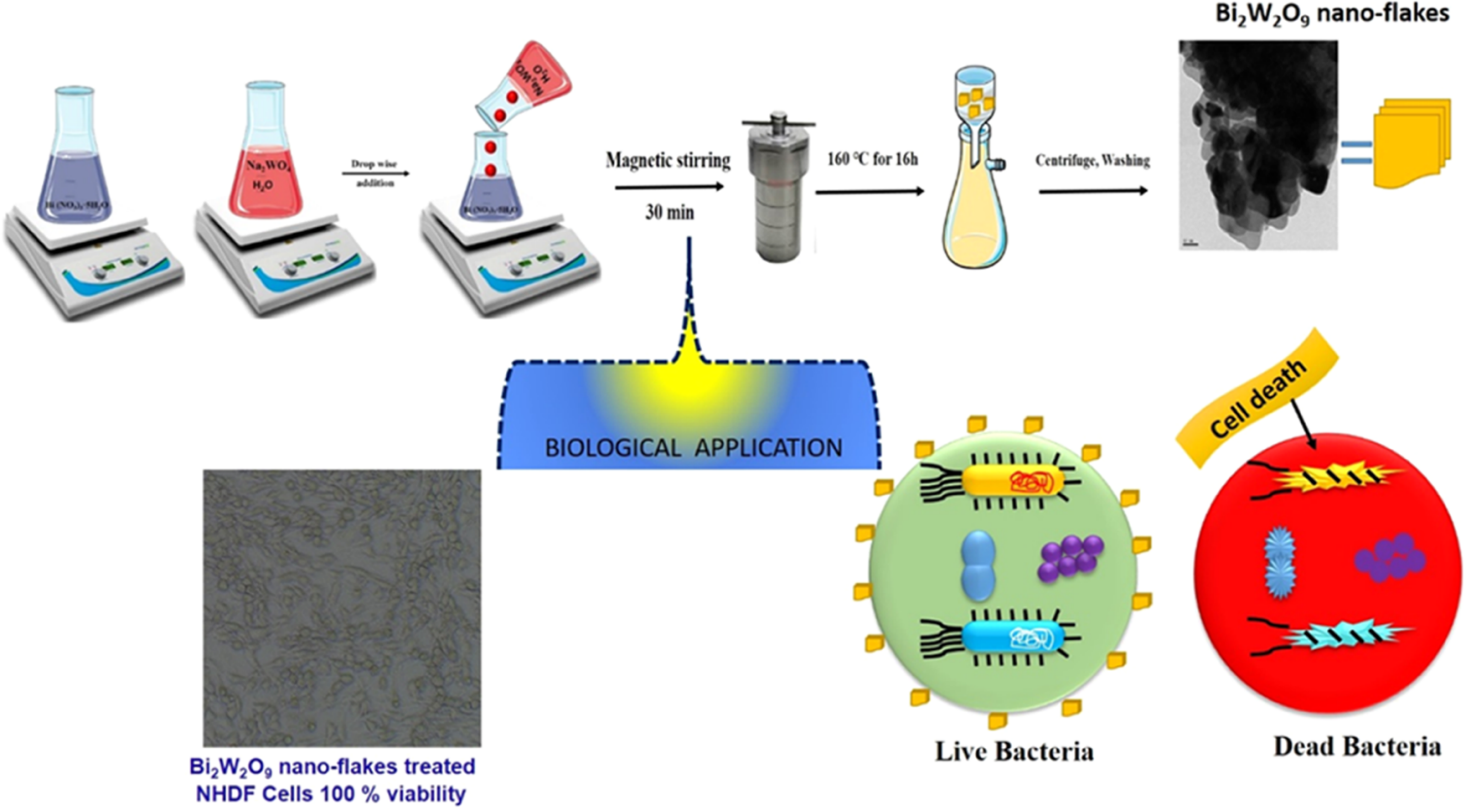
Hydrothermal Process of Bi_2_W_2_O_9_ Formation and Its Biomedical Application

The concentration of the sample was represented along the *X*-axis, and the percentage of viable cells was utilized to generate the graphs. To compare the whole cell viability evaluations, both the cell regulator and sample regulator were included in each test (Scheme 1).

Viable and nonviable cells were labeled using fluorescence imaging and calcein/PI dual labeling in order to enhance understanding of the destructive effects of Bi_2_W_2_O_9_ nanoflakes on NHDF cells. Glass bottom plates were used to culture NHDF cells until they achieved 70% confluence. Following that, they were subjected to Bi_2_W_2_O_9_ nanoflakes for 24 and 48 h. The cells were dyed for 30 min at 37 °C in a dark incubator. After that, they underwent two PBS washes, and a confocal fluorescence microscope (Stellaris 5WLL confocal scanning system) was used for observation.

## 3 Result and Discussion

### 3.1 XRD Investigation

**Figure 1:**
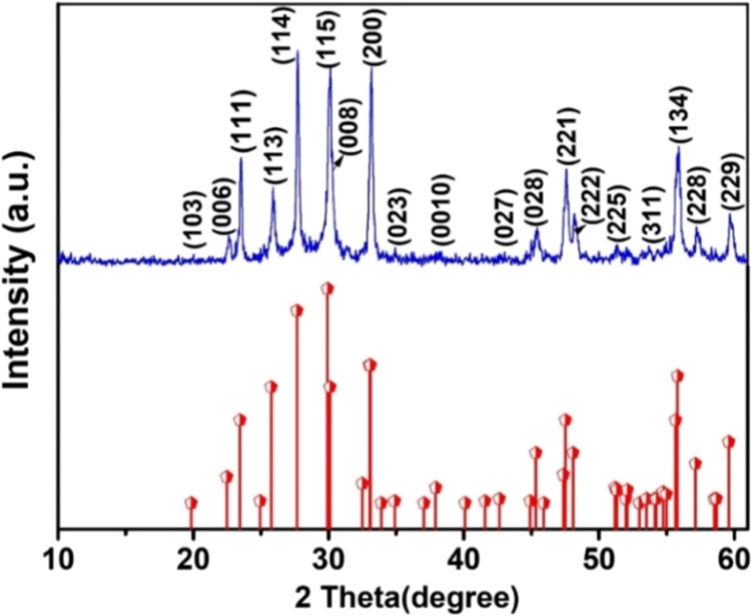
X-ray diffraction of Bi_2_W_2_O_9_ nanoflakes.

The XRD pattern displayed in Figure 1 illustrates the synthesized Bi_2_W_2_O_9_, with all diffraction peaks corresponding to the monoclinic configuration of Bi_2_W_2_O_9_ in space group p2/c of JCPDS Card No 00–033–0221. The peaks are associated with (103), (006), (111), (114), (115), (008), (200), (023), (0010), (027), (028), (221), (222), (225), (311), (134), (228), and (229) planes of orthorhombic Bi_2_W_2_O_9_. The lattice parameters of Bi_2_W_2_O_9_ are *a* = 5.413 Å, *b* = 5.413 Å, and *c* = 23.694 Å, with α = β = γ. The generated Bi_2_W_2_O_9_ has noticeable diffraction peaks, which suggest that the material is highly crystalline. No other peaks of contamination were discovered.^23^

### 3.2 XPS Analysis of Bi_2_W_2_O_9_ Nanoflakes

**Figure 2:**
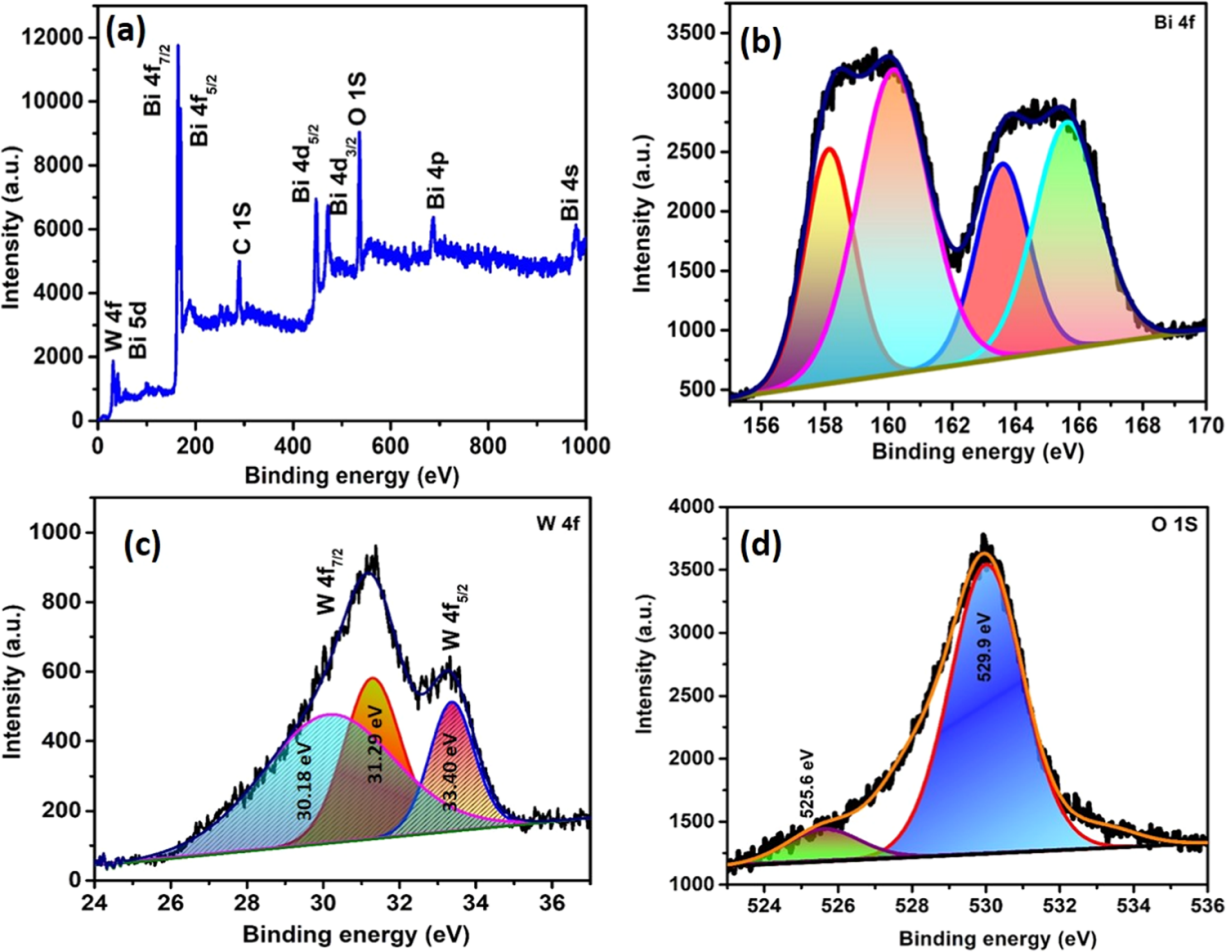
(a) XPS survey spectrum of Bi_2_W_2_O_9_ nanoflakes; (b) the core level spectrum of Bi; (c) the core level spectrum of W; and (d) the core level spectrum of O.

The element’s oxidation states, binding energies, and chemical nature in Bi_2_W_2_O_9_ were confirmed through XPS analysis. The survey XPS spectrum of Bi_2_W_2_O_9_ is presented in Figure 2a, revealing the presence of Bi, W, and O in the produced material. This finding aligns with the results obtained from the EDAX analysis. Further discussion on the high-resolution XPS spectra of Bi 4f and W 4f in the synthesized Bi_2_W_2_O_9_ nanoparticles will follow. In Figure 2b, the XPS spectrum of Bi 4f is depicted, revealing the presence of four distinct peaks. These peaks can be attributed to Bi 4f 7/2 and Bi 4f 5/2, which are observed at energy levels of 158 and 163 eV, respectively, which belong to the +3 oxidation state of Bi. Additionally, there are peaks observed at 160 and 165 eV; these two peaks at higher binding energy among the four deconvoluted peaks can be attributed to a slight surface charging effect from the polarization change in the crystal.^24,25^Figure 2c displays the XPS spectrum of W 4f, exposing two peaks corresponding to W 4f 7/2 with two splits at 30.18 eV and 31.29. W 4f 5/2 at 33.40 eV, respectively, with the fitting of three peaks. The detection of the spin–orbit twins of W 4f 7/2 and W 4f 5/2 at 2.2 eV indicates that W is in the +6 oxidation state.^26^ The O 1s peak clearly displays the main component, which is centered at 529.9 eV with high binding energy and 525.6 eV with low binding energy. The peak at 529.9 eV belongs to Bi–O in synthesized Bi_2_W_2_O_9_. The lower binding energy peak located at 525.6 eV corresponds to lattice oxygen within the Bi_2_W_2_O_9_ framework. Lattice oxygen atoms are bound within the crystalline structure of metal oxides such as tungsten oxide in the compound. The lower binding energy (5) indicates oxygen atoms that are part of the Bi–O bonds in the perovskite-like structure of Bi_2_W_2_O_9_.^27^

### 3.3 Raman of Bi_2_W_2_O_9_ Nanoflakes

**Figure 3:**
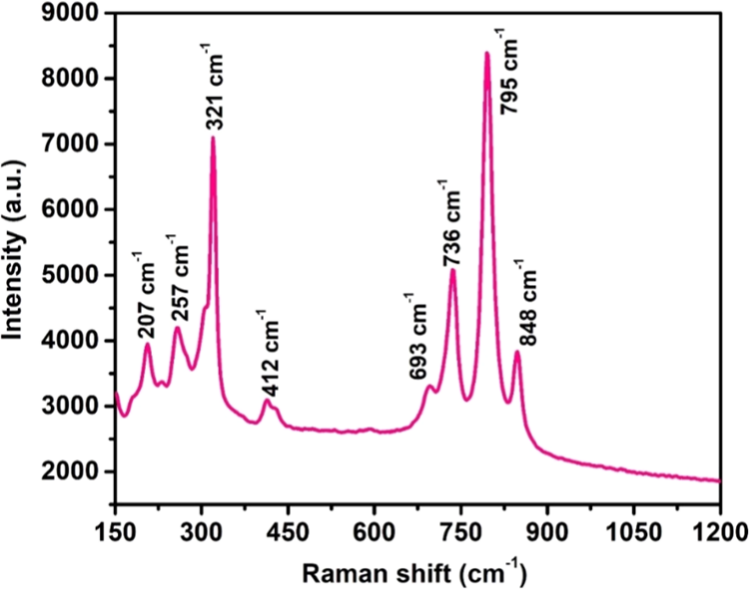
Raman spectrum of Bi_2_W_2_O_9_ nanoflakes.

In Figure 3, the synthesized Bi_2_W_2_O_9_ Raman spectrum is shown. For the synthesized Bi_2_W_2_O_9_, a wide range of modes, including symmetric, asymmetric stretching, bending, scissoring, twisting, and translational modes, are represented by the bands seen in the spectrum. The bands that were detected for Bi_2_W_2_O_9_ were 848, 795, 736, 693, 412, 321, 257, and 207 cm^–1^. These bands are the result of distinct absorption modes at various translational levels. The severe asymmetric stretching of the (W–O) group in the Bi_2_W_2_O_9_ compound is shown by an extremely intense band at 848 cm^–1^.^28^ The bands detected at 795 and 736 cm^–1^ suggest the existence of symmetric and asymmetric stretching vibration modes of the (W–O) bond.^29^ The 693 cm^–1^ peak is allocated to the stretching vibration of W–O–W, while the 412 cm^–1^ band is indicative of the presence of Bi_2_O_3_.^30^ The medium scissoring of both WO_2_ and W–O–W is confirmed by the band at 321 cm^–1^.^31^ The bending mode of [WO_6_]^6–^ and the twisting mode of the WO_2_ group are suggested by a band seen at 257 cm^–1^. Furthermore, the bands at 207 cm^–1^ suggest that a Bi–O bond may exist.^30^

### 3.4 FTIR Analysis

**Figure 4:**
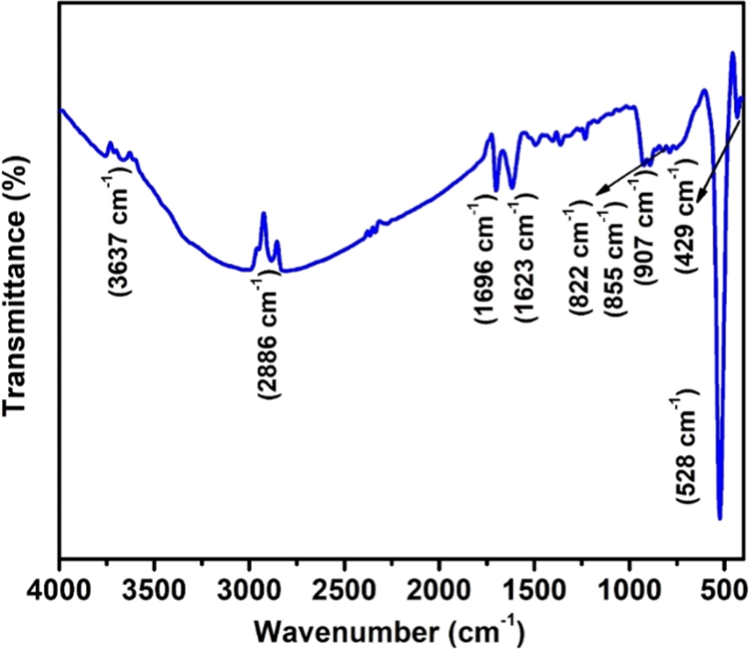
FTIR spectrum of Bi_2_W_2_O_9_ nanoflakes.

The FTIR spectrum depicting synthesized Bi_2_W_2_O_9_ can be seen in Figure 4. A wide band detected at 3637 cm^–1^ suggests the vibration of the O–H bond due to surface hydration. The symmetric stretching of the −CH_2_– groups in the structure of SDS was assigned to the strong bands at 2923 and 2886 cm^–1^, respectively.^32^ Additionally, the bands at 1696 and 1623 cm^–1^ are attributed to the H–O–H deformation vibration of the surface hydroxyl group.^33^ The stretching modes of Bi–O, W–O, and W–O–W are responsible for the absorption peaks observed at 907, 528, and 429 cm^–1^, respectively.^34^ The stretching vibrations of the Bi–O bond and the W–O bond are observed at 822 and 855 cm^–1^, respectively, in the week band.^35^

### 3.5 Analysis of UV–Visible Spectra Using the DRS Technique

**Figure 5:**
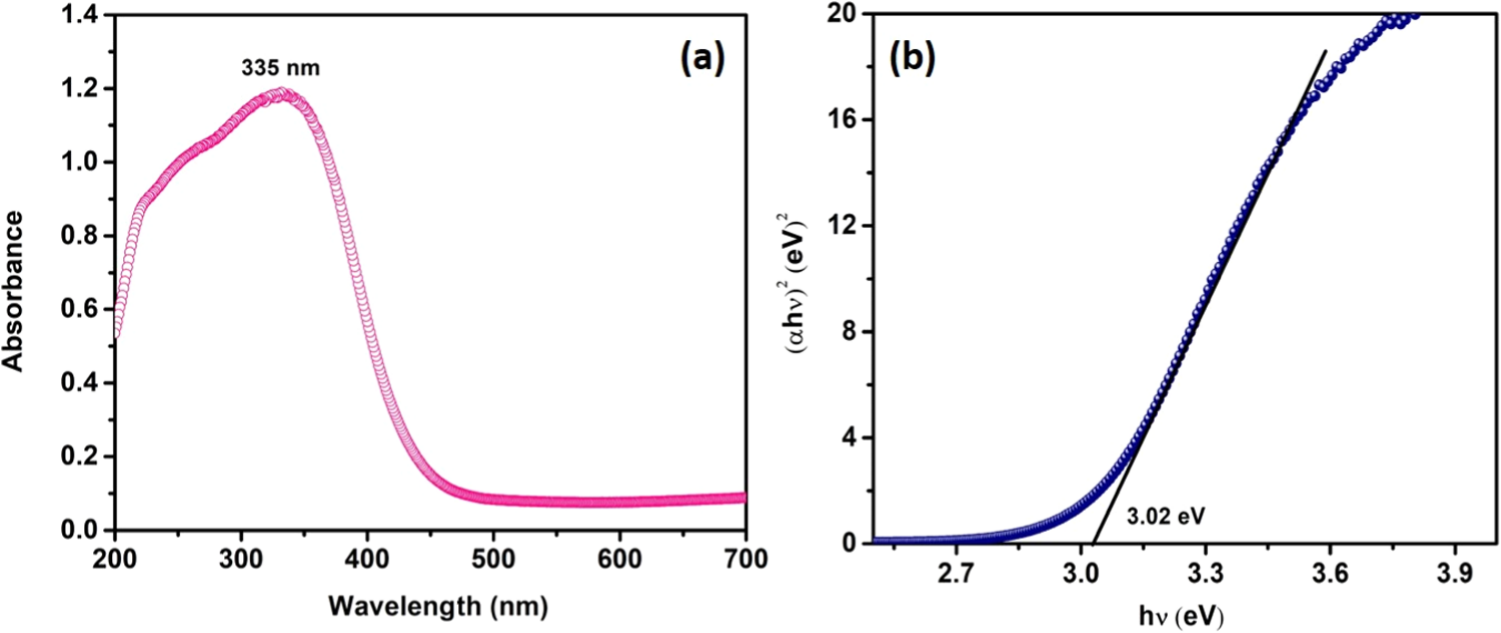
Panel (a) shows the UV spectrum of Bi_2_W_2_O_9_ nanoflakes, while panel (b) presents Tauc’s plot.

The DRS-UV spectroscopy was employed to analyze the optical characteristic of the synthesized Bi_2_W_2_O_9_, and the corresponding spectrum can be observed in Figure 5a. The absorption peak was observed within the range of 280 to 350 nm. The band gap significance (*E*_g_) of the synthesized Bi_2_W_2_O_9_ was determined through the utilization of Tauc’s plot.
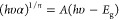


Figure 5b, inset, shows Tauc’s plot of Bi_2_W_2_O_9_, with a direct band gap of 3.02 eV.^36^ It can be seen that the Bi_2_W_2_O_9_ nanoparticle possesses a larger band gap (2.68 eV) than bulk-WO_3_.^37^ The quantum size confinement of the W_2_O_6_ levels in Bi_2_W_2_O_9_ must be responsible for at least some of the band gap increase.

### 3.6 Structural Examination

**Figure 6:**
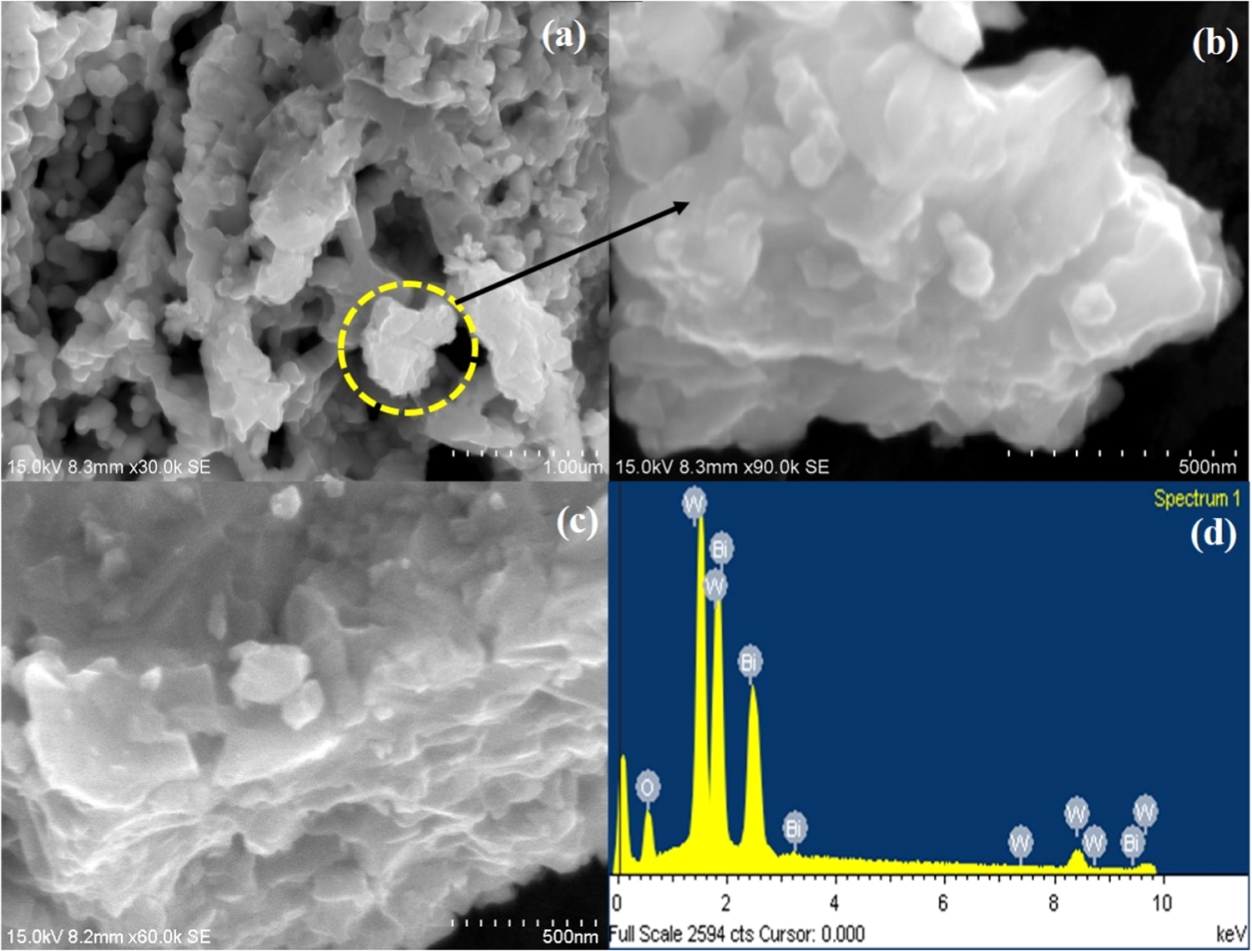
(a–c) FE-SEM images with different magnification, and (d) EDAX of Bi_2_W_2_O_9_ nanoflakes, respectively.

**Figure 7:**
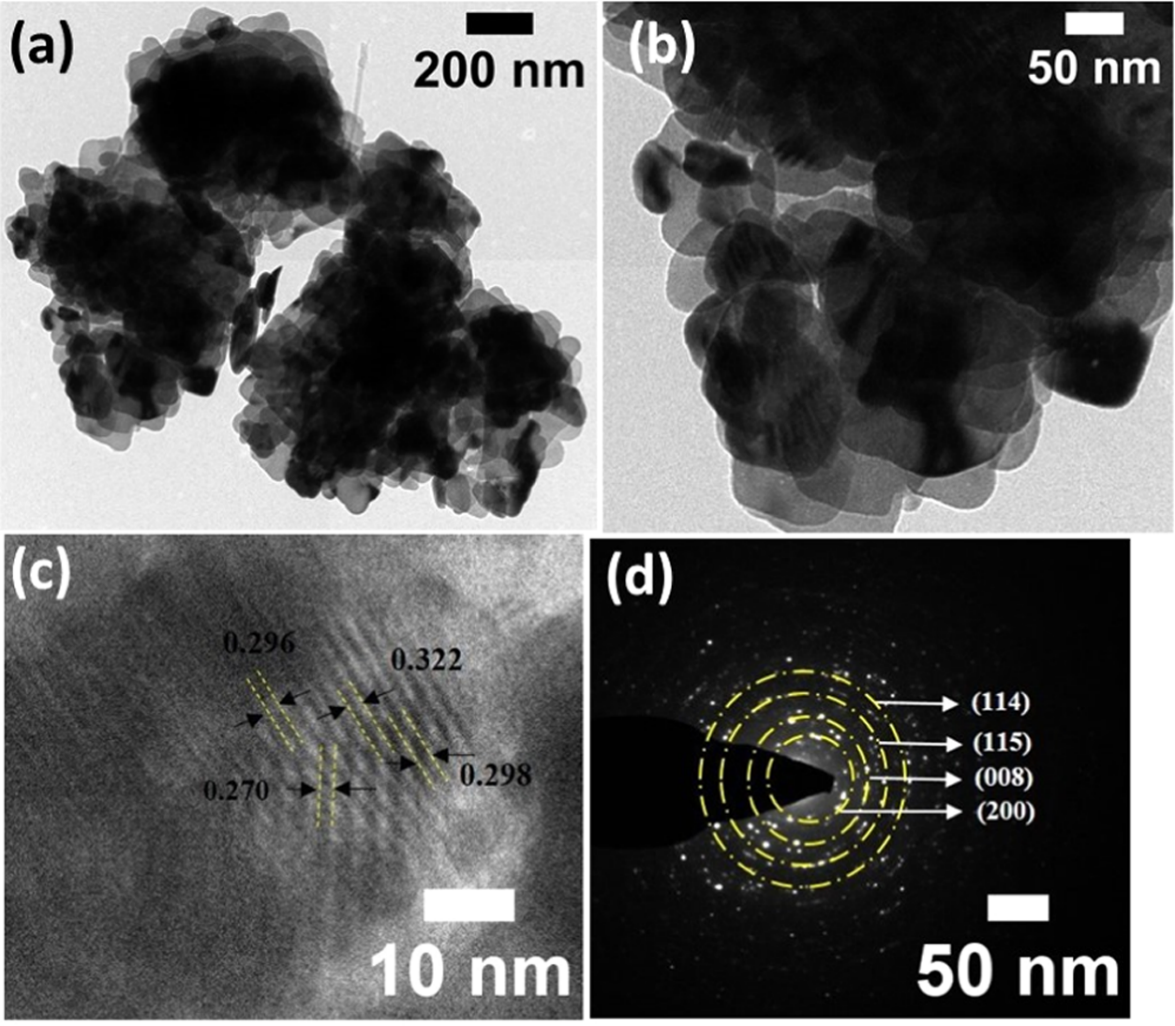
(a, b) HR-TEM images of Bi_2_W_2_O_9_ at different magnifications, along with (c) fringe patterns and (d) the SAED pattern.

Figure 6a–c displays the FE-SEM image of Bi_2_W_2_O_9_. FE-SEM images make it abundantly evident that the produced Bi_2_W_2_O_9_ had a flake-like shape. Multiple layers are stacked together and formed as flake-like morphology.^38^Figure 6a–c shows the different magnifications of bismuth tungstate flakes of 1 μm and 500 nm. Bi, W, and O are present as predicted according to the matching EDAX spectrum (Figure 6d), and no additional contaminants were found. HR-TEM examination was conceded to endorse the flake-like structure of bismuth tungstate. HR-TEM pictures of Bi_2_W_2_O_9_ are revealed in Figures 7a,b and S1. From the observed results it is clearly evident that transparent staked layers are present in the compound with nanometers in the scale. Figure 7c displays the fringe pattern of the Bi_2_W_2_O_9_ done on the randomly selected particle at the magnification of 10 nm; the space between the selected fringes is 0.322 and 0.270 nm. Additionally, as seen in Figure 7d, the selected area electron diffraction (SAED) pattern displays a regular diffraction spot array with a spacing of 0.322, 0.298, 0.296, and 0.270 nm, which correspond to the (114), (115), (008), and (200) planes, respectively. The above observation evidently specifies that the Bi_2_W_2_O_9_ was shown to have a highly crystalline character using XRD examination.

## 4 Biological Application of Synthesized Bi_2_W_2_O_9_ Nanoflakes

### 4.1 Antimicrobial Activity

Bi_2_W_2_O_9_ nanoflakes were tested at 50, 100, and 150 μg/mL for their antibacterial activity against strains of *E. coli*, *E. faecalis*, *S. aureus*, and *P. aeruginosa* bacteria. Bi_2_W_2_O_9_ nanoflakes exhibited substantial antibacterial activity against both bacterial strains. When *E. coli*, *P. aeruginosa*, *E. faecalis*, and *S. aureus* were at high concentrations, the inhibition zones were about 15 ± 0.16, 17.2 ± 0.85, 10.53 ± 0.63, and 16.4 ± 0.32 mm, respectively, at 150 μg concentration.

**Figure 8:**
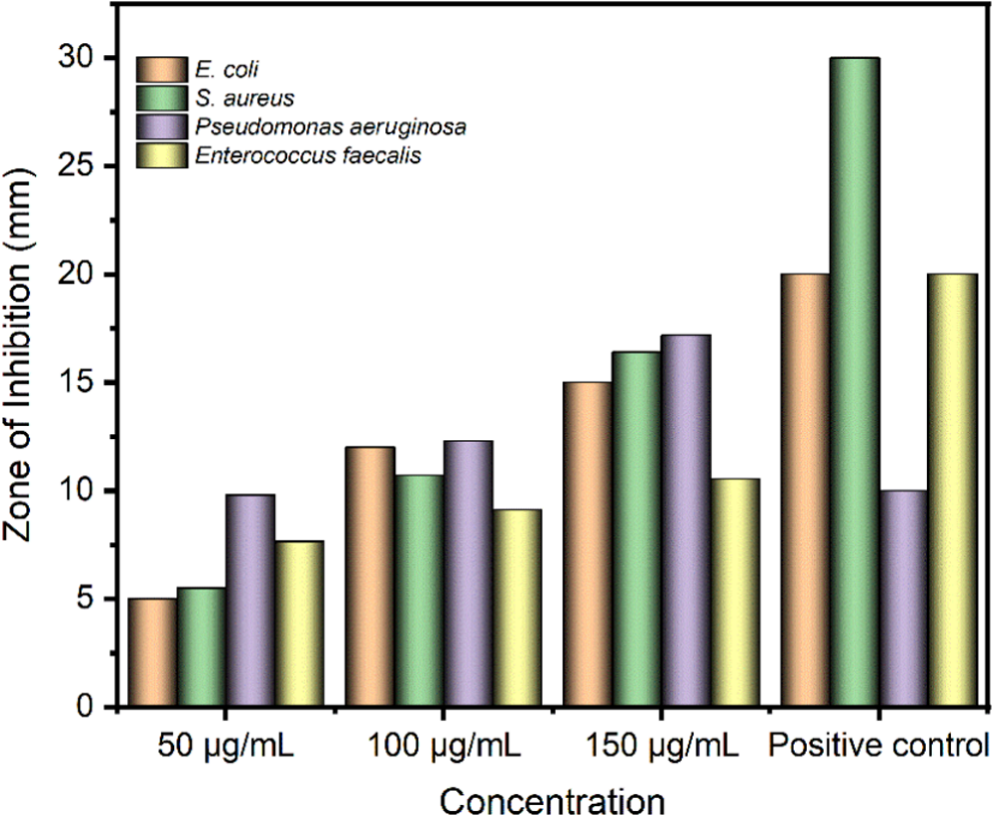
Antibacterial activity of Bi_2_W_2_O_9_ nanoflakes at different concentrations against oral pathogens.

The study revealed that a concentration of 150 μg/mL induced the greatest zone of inhibition in *E. coli*. However, even at the lowest concentration of 50 μg/mL, minimal activity was observed in *E. coli* (Figures 8 and S3), and a comparable trend was noted in *P. aeruginosa*. On the other hand, *S. aureus* exhibits a significant augmentation in the zone of inhibition when exposed to a dose of 100 μg/mL, followed by a marginal increase at a dosage of 150 μg/mL. This observation suggests that *S. aureus* exhibited a higher susceptibility to Bi_2_W_2_O_9_ nanoflakes or that a point of equilibrium in the antibacterial efficacy was attained within the concentration range of 100 to 150 μg/mL. Conversely, *E. faecalis*, akin to *P. aeruginosa* and *E. coli*, exhibited expanded zones of inhibition at elevated concentrations. The findings of this investigation indicate that both *E. coli* and *P. aeruginosa* exhibited a comparable trend of progressive expansion in the zone of inhibition as the concentration of the nanoflakes increased. These observations may be attributed to the fact that both bacteria are Gram-negative and possess an outer membrane that potentially restricts the infiltration of the nanoflakes.^39,40^ Consequently, larger quantities of nanoflakes may be necessary to achieve substantial antibacterial efficacy. On the other hand, two Gram-positive bacteria, *S. aureus* and *E. faecalis*, show distinct patterns of susceptibility to Bi_2_W_2_O_9_ nanoflakes. *E. faecalis* exhibits a notable augmentation in sensitivity when exposed to the nanoflakes at the greatest concentration, suggesting a potential heightened susceptibility or the existence of a threshold beyond which the antibacterial efficacy abruptly intensifies.^41,42^ These findings have the potential to be significant in the advancement of novel antibacterial therapies, particularly if the nanoflakes possess distinctive characteristics that might be advantageous for oral administration.

Our findings indicate that the antibacterial properties of the nanostructures are significantly influenced by their size, surface morphology, and the specific bacterial strains tested. Additionally, we observed that the inhibitory effect of Bi_2_W_2_O_9_ increased with higher concentrations. The results further suggest that the enhanced antibacterial activity of Bi_2_W_2_O_9_ is associated with the increased permeability of the bacterial membrane, which facilitates the entry of the abrasive-textured nanostructures. This interaction results in membrane disruption and alterations at the protein level, ultimately leading to bacterial cell death due to the inhibition of biological metabolism.

### 4.2 Minimum Inhibitory Concentration

The results of the Minimum Inhibitory Concentration (MIC) assay indicate that Bi_2_W_2_O_9_ nanoflakes exhibit antibacterial activity against *E. coli*, *E. faecalis*, and *S. aureus* (Table 1), while they show no efficacy against *P. aeruginosa*. Specifically, the MIC for both *E. coli* and *E. faecalis* is established at 75 μg/mL, as bacterial inhibition commences at this concentration and remains effective at lower concentrations of 50 and 25 μg/mL. For *S. aureus*, the MIC is determined to be 25 μg/mL, with inhibition observed solely at this concentration. In contrast, *P. aeruginosa* demonstrates no inhibition at any of the tested concentrations, indicating a state of resistance. Notably, the positive control also exhibited no inhibition, suggesting a potential experimental issue with the control conditions.^43,44^ Furthermore, Bi_2_W_2_O_9_ nanoflakes show promise as an antibacterial agent against specific bacterial strains. They exhibit a comparable or slightly elevated MIC for Gram-positive bacteria (*E. faecalis* and *S. aureus*) in comparison to silver or zinc oxide nanoparticles. This enhanced activity may be attributed to a greater surface area and the release of a higher concentration of ions with significant antimicrobial activity relative to nanoparticles of other morphologies.^45^

**Table 1:** Minimum Inhibitory Concentration (MIC), Turbidity for Different Concentrations of Bi_2_W_2_O_9_ Nanoflakes after 24 h^a^

	dilution of Bi_2_W_2_O_9_ nanoflakes (μg/mL)						
pathogenic bacteria	150	100	75	50	25	PC	NC
*E. coli*	–ve	–ve	+ve	+ve	+ve	–ve	+ve
*E. faecalis*	–ve	–ve	+ve	+ve	+ve	–ve	+ve
*S. aureus*	–ve	–ve	–ve	–ve	+ve	–ve	+ve
*P. aeruginosa*	–ve	–ve	–ve	–ve	+ve	–ve	+ve

a Positive (+): Turbidity indicating growth. Negative (−): No turbidity indicating absence of growth.

### 4.3 Effect of Bi_2_W_2_O_9_ on Cell Viability

**Figure 9:**
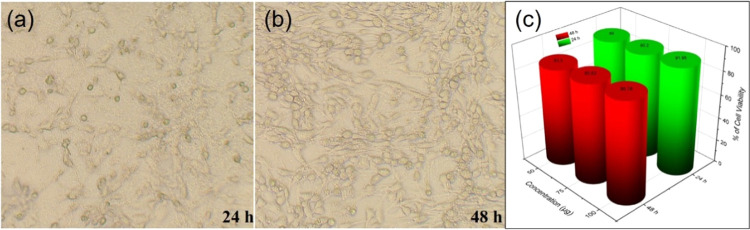
Effect of Bi_2_W_2_O_9_ nanoflakes on NHDF cell lines. (a) 24 h treatment; (b) 48 h treatment; and (c) MTT-based colorimetric cytotoxic assay.

**Figure 10:**
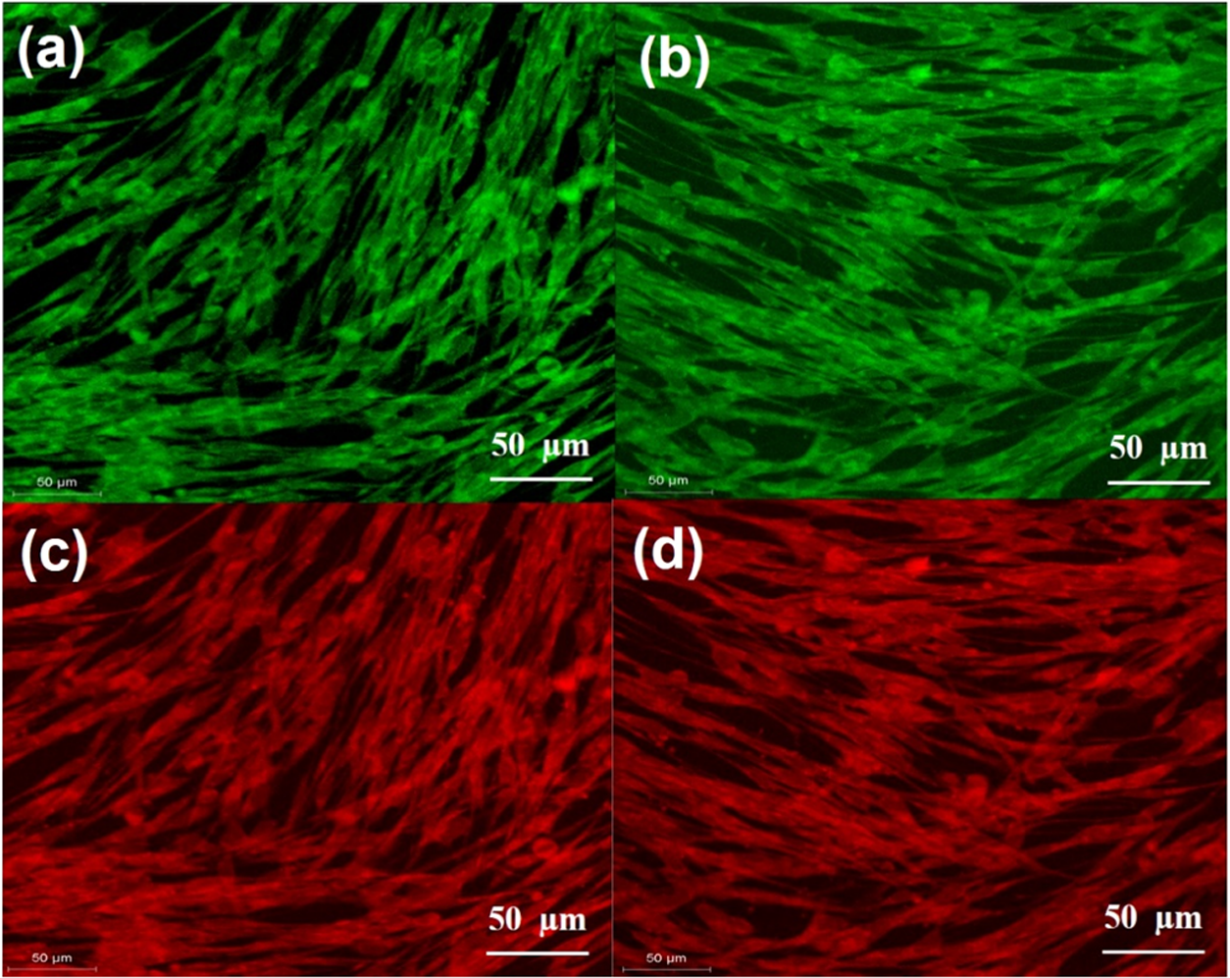
(a–d) Confocal fluorescence microscopy images of the cell viability and morphology.

In this study, the cell viability and cytotoxicity of the Bi_2_W_2_O_9_ nanoflakes were examined on normal human dermal fibroblast (NHDF) cell lines using different nanoflake concentrations such as 50, 75, and 100 μg/mL for between 24 and 48 h, Figure 9a–c. The morphology of the cells appears relatively normal without signs of cytotoxicity at 24 h. After 48 h, the NHDF cells appeared to have increased confluence, and there may be some morphological changes indicative of cellular responses to the treatment. However, the cells showed clear signs of no cytotoxicity, such as cell rounding or detachment. The confocal images depicting NHDF cell lines after 24 and 48 h of exposure to Bi_2_W_2_O_9_ nanoflakes often depict viable cells, Figure 10a–d. Both periods exhibit elongated, fibrous structures in the green fluorescence image of cells, suggesting a well-preserved cytoskeleton. Significant mitochondrial activity and cell membrane integrity were seen in the red channel. Cells exhibit elongated structures at both periods, indicating the presence of healthy mitochondrial networks or intact cell membranes similar to those in the green channel. The constant presence of Bi_2_W_2_O_9_ nanoflakes at both periods indicates that they have no substantial impact on cell viability throughout the studied period. The cellular shape seen in this study seems to be consistent with the results, suggesting that the Bi_2_W_2_O_9_ nanoflakes used in this study may fall within a biocompatible range. Besides, the in vitro MTT assay depicts that the cell viability remains high at 24 h as 89, 90.2, and 91.95% for 50, 75, and 100 μg, respectively, Figure 9c, indicating low cytotoxicity at these concentrations and exposure time. In contrast, a slight decrease in cell viability at 48 h was observed at all concentrations. The present result suggests a dose-dependent cytotoxic effect, which is more pronounced with longer exposure. The observed cytotoxicity is relatively low, considering that the viability is above 80% for all tested concentrations and times. This suggests that Bi_2_W_2_O_9_ nanoflakes might have a tolerable profile in NHDF cells at these concentrations. This is typically low in cytotoxicity to NHDF cells at these concentrations, which is consistent with previous reports.^46−48^

## 5 Conclusions

In summary, the one-step hydrothermal method was effective in synthesizing Bi_2_W_2_O_9_ nanoflakes. The Bi_2_W_2_O_9_ nanoflakes that were produced had an average size of around 20 nm. Additionally, by showing the existence of significant indications of Bi, W, and O, the EDX-based elemental analysis of the nanoflakes has corroborated their elements. Through the properties of the Miller indices and diffraction peak planes of the Bi_2_W_2_O_9_ nanoflakes, the XRD data further proved their crystalline nature and bimetallic composition. Bi_2_W_2_O_9_ nanoflakes have demonstrated encouraging antibacterial properties in vitro against strains of *S. aureus*, *P. aeruginosa*, *E. faecalis*, and *E. coli*. This investigation examined the cytotoxicity and cell survival of Bi_2_W_2_O_9_ nanoflakes on normal human dermal fibroblast (NHDF) cell lines over the course of 24 to 48 h at various nanoflake concentrations, including 50, 100, and 150 μg/mL. After 24 h, the cell shape seems to be mostly normal and there are no indications of cytotoxicity. Overall, it is hoped that the study’s findings will open up a number of novel avenues for the development of Bi_2_W_2_O_9_ as prospective multifunctional nanoformulations in the fields of nanobiotechnology and nanomedicine.

## Supporting Information Available

HR-TEM of Bi_2_W_2_O_9_ nanoflakes (Figure S1); BET surface analysis of the nanoflakes were analyzed (section 2 and Figure S2); zone of inhibition of synthesized Bi_2_W_2_O_9_ nanoflakes against *E. coli*, *E. faecalis*, and *S. aureus*, and *P. aeruginosa* bacteria images (Figure S3) (PDF)
