# Supplementary material for: Bi2W2O9 Nanoflakes Synthesized via a Hydrothermal Method: Antibacterial Potency and Cytotoxicity Evaluation on Human Dermal Fibroblasts
Source: ACS Omega. 2025 Feb 5;10(6):5468–77. doi: 10.1021/acsomega.4c07612 (PMC11840603; doi:10.1021/acsomega.4c07612)
Supplement: Supplementary file 1 — ao4c07612_si_001.pdf [file ao4c07612_si_001.pdf]

$Bi_2W_2O_9$ Nanoflakes Synthesized via Hydrothermal Method: Antibacterial Potency and Cytotoxicity Evaluation on Human Dermal Fibroblasts

Muthamizh Selvamani[a], Dilipan Elangovan[a]*, Ali Alsalme [b] Arul Varman Kesavan[c] * Ganeshraja Ayyakannu Sundaram[d], A. Santhana Krishna Kumar [e, f] *

[a]Department of Physiology, Saveetha Dental College & Hospitals, Saveetha Institute of Medical & Technical Sciences, Saveetha University, Chennai - 600077, Tamil Nadu, India.

[b]Department of Chemistry, College of Science, King Saud University, Riyadh 11451, Saudi Arabia.

[c]Department of Physics & Nanotechnology, SRM Institute of Science & Technology, Kattankulathur-603203, Tamil Nadu, India.

[d]Department of Research Analytics, Saveetha Dental College and Hospitals, Saveetha Institute of Medical and Technical Sciences, Poonamallee High Road, Chennai 600 077, 15 Tamil Nadu State, India;

[e]Department of Chemistry, National Sun Yat-sen University, No. 70, Lien hai Road, 17 Gushan District, Kaohsiung 80424, Taiwan.

[f]Department of Chemistry, Saveetha School of Engineering, Saveetha Institute of Medical and Technical Sciences (SIMATS), Saveetha University, Chennai, Tamil Nadu 602 105, India.

Contact correspondence to:

Dr. A. Santhana Krishna Kumar *

Department of Chemistry, National Sun Yat-sen University, No. 70, Lien-hai Road, Gushan District, Kaohsiung City-80424, Taiwan.

Department of Chemistry, Saveetha School of Engineering, Saveetha Institute of Medical and Technical Sciences (SIMATS), Saveetha University, Chennai, Tamil Nadu 602 105, India.

E-mail: krishnakumar@mail.nsysu.edu.tw

Elangovan Dilipan

Department of Physiology, Saveetha Dental College & Hospitals, Saveetha Institute of Medical & Technical Sciences, Saveetha University, Chennai - 600077, Tamil Nadu, India.

E-mail: gerberadilip@gmail.com.

Arul Varman Kesavan

Department of Physics & Nanotechnology, SRM Institute of Science & Technology, Kattankulathur-603203, Tamil Nadu, India.

E-mail id: arulkvarman@gmail.com

1. **HR-TEM of $Bi_2W_2O_9$ nanoflakes**

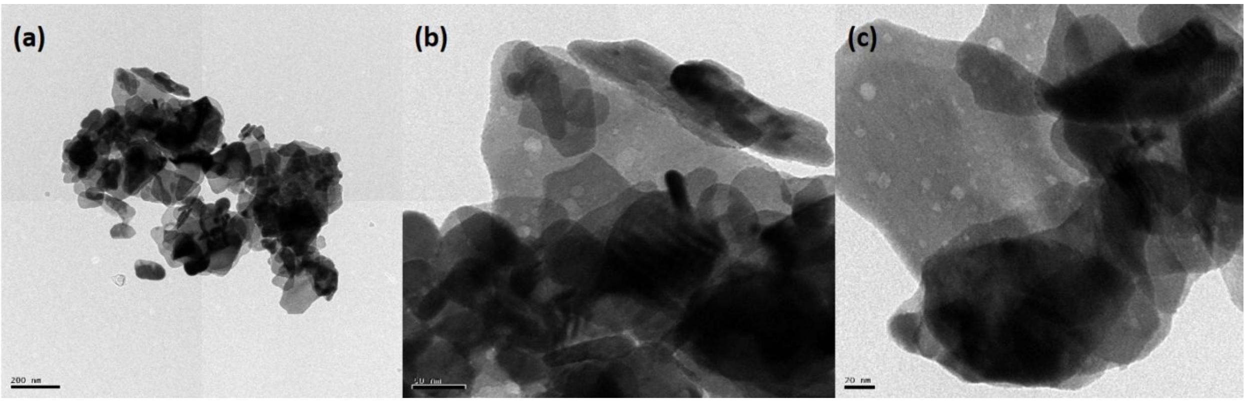

Figure S1. (a-c) HR-TEM images of $Bi_2W_2O_9$ nano flakes.

2. **BET surface area of $Bi_2W_2O_9$ nanoflakes.**

By physically adsorbing and desorbing N_2 gas, the surface area of $Bi_2W_2O_9$ nanoflakes was determined using the Brunauer-Emmett-Teller (BET) analytical method. Obtained a result in the range of 0 to 1.2 relative pressure for the produced material. Based on the IUPAC classification, the adsorption isotherm has a specific surface area of 23.59 m^2/g, which indicates that it is type IV. The result is shown in **Figure S2.** Due to the material has high surface area and suitable porosity, it can offer more active sites for biological interactions which favors the antibacterial property.

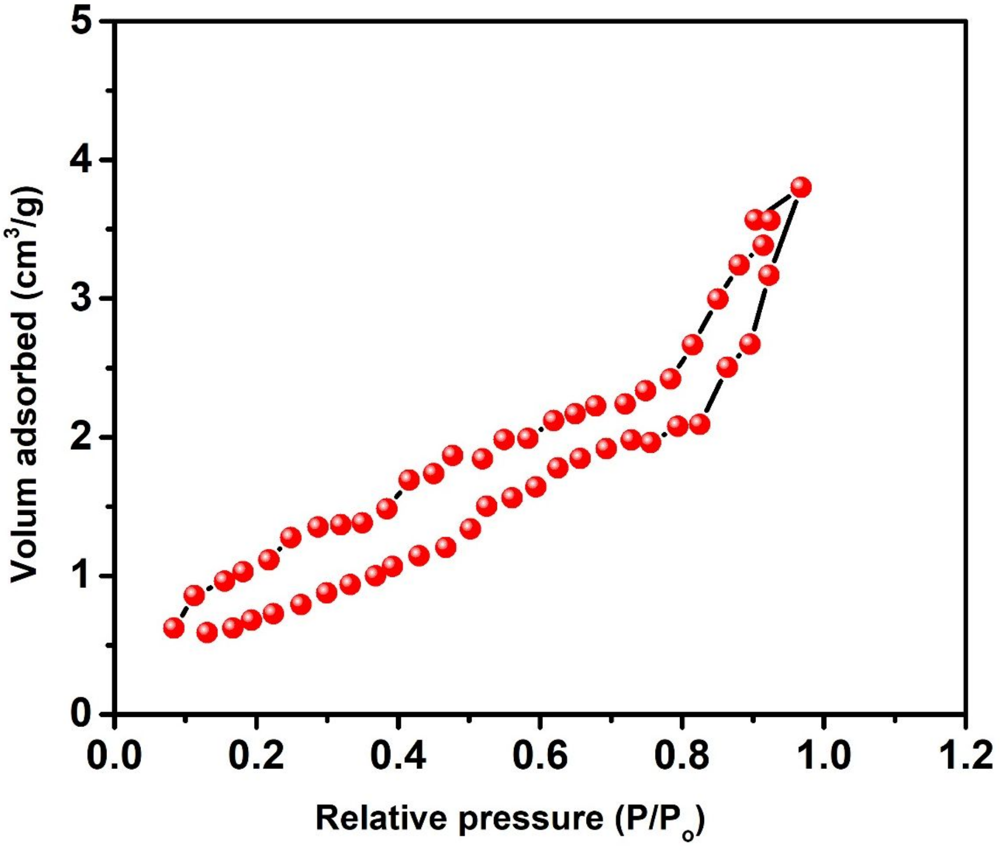

Figure S2. Isotherms of the synthesized $Bi_2W_2O_9$ nanoflakes.

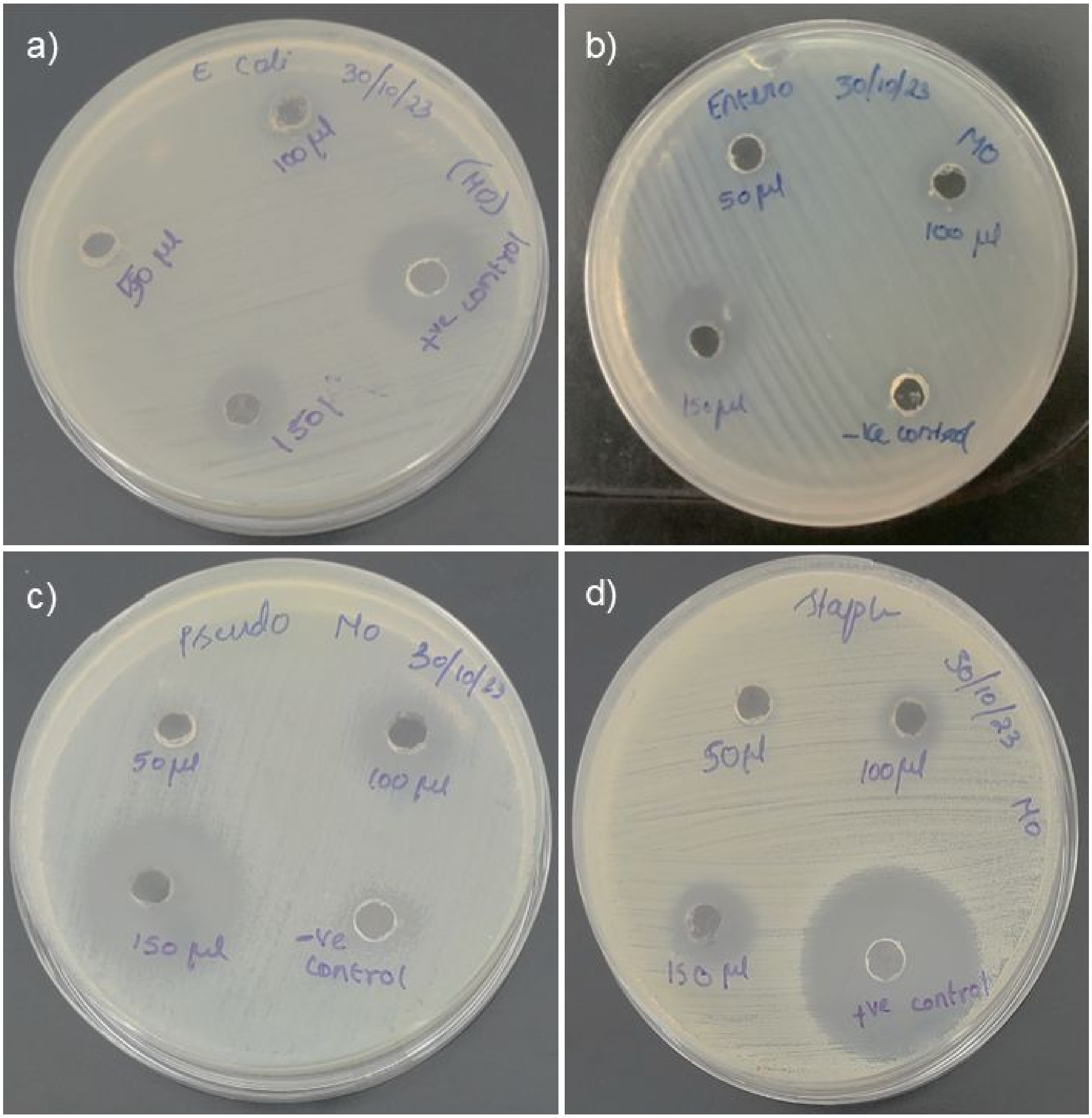

Figure S3. The zone of inhibition of synthesized $Bi_2W_2O_9$ nano flakes against (a) *Escherichia coli*, (b) *Enterococcus faecalis*, (c) *Staphylococcus aureus*, and (d) *Pseudomonas aeruginosa* bacteria.

Table S1. Comparison of the MIC values of various bismuth nanomaterials against pathogenic bacteria

Material	MIC (µg mL^{-1})				Reference
	E. coli	*E. faecalis*	*S. aureus*	*P. aeruginosa*	
Bi/Ag NPs	3.44	-	1.72	-	S1
Bi_2O_3 NP	-	-	128	-	S2
Bi_2O_3 nano-flakes	23.3	-	26.7	-	S3
BiNPs	-	1.25	-	-	S4
Bismuth Nanoparticle	-	>1000	>1000	>1000	S5
Bi2S3NPs	164	-	329	164	S6
BiNPs	267	-	267	267	S6
Bi_2O_3NPs	267	-	267	149	S6
$BiFeO_3$ nanoparticles	>100	-	>100	-	S7

References:

1. Castro-Valenzuela BE, Franco-Molina MA, Zárate-Triviño DG, Villarreal-Treviño L, Kawas JR, García-Coronado PL, Sobrevilla-Hernández G, Rodríguez-Padilla C. Antibacterial efficacy of novel bismuth-silver nanoparticles synthesis on Staphylococcus aureus and Escherichia coli infection models. Frontiers in Microbiology. 2024 Apr 8;15:1376669. doi.org/10.3389/fmicb.2024.1376669
2. Jawad KH, Marzoog TR, Hasoon BA, Sulaiman GM, Jabir MS, Ahmed EM, Khalil KA. Antibacterial activity of bismuth oxide nanoparticles compared to amikacin against acinetobacter baumannii and Staphylococcus aureus. Journal of Nanomaterials. 2022;2022(1):8511601. https://doi.org/10.1155/2022/8511601
3. Geoffrion LD, Medina-Cruz D, Kusper M, Elsaidi S, Watanabe F, Parajuli P, Ponce A, Hoang TB, Brintlinger T, Webster TJ, Guisbiers G. Bi 2 O 3 nano-flakes as a cost-effective antibacterial agent. Nanoscale Advances. 2021;3(14):4106-18. DOI: 10.1039/D0NA00910E
4. Azad A, Rostamifar S, Modaresi F, Bazrafkan A, Rezaie Z. Assessment of the antibacterial effects of bismuth nanoparticles against Enterococcus faecalis. BioMed Research International. 2020;2020(1):5465439. https://doi.org/10.1155/2020/5465439
5. Huang R, Zhou Z, Lan X, Tang FK, Cheng T, Sun H, Leung KC, Li X, Jin L. Rapid synthesis of bismuth-organic frameworks as selective antimicrobial materials against microbial biofilms. Materials Today Bio. 2023 Feb 1;18:100507. https://doi.org/10.1016/j.mtbio.2022.100507
6. Campos V, Almaguer-Flores A, Velasco-Aria D, Díaz D, Rodil SE. Bismuth and silver nanoparticles as antimicrobial agent over subgingival bacterial and nosocomial strains. J Mater Sci Eng A. 2018;8(7-8):142-6. doi: 10.17265/2161-6213/2018.7-8.002
7. Pulvirenti L, Lombardo C, Salmeri M, Bongiorno C, Mannino G, Lo Presti F, Cambria MT, Condorelli GG. Self-assembled BiFeO3@ MIL-101 nanocomposite for antimicrobial applications under natural sunlight. Discover Nano. 2023 Sep 11;18(1):113. https://doi.org/10.1186/s11671-023-03883-9
